# Artificial Intelligence in Emergency Medical Call-Taking and Dispatch Operations: A Scoping Review

**DOI:** 10.3390/healthcare14193176

**Published:** 2026-09-24

**Authors:** Da Hye Lee, Min Joung Kim, Hyuk-Jae Chang

**Affiliations:** 1 University-Industry Foundation, Yonsei University Health System, Seoul 03722, Republic of Korea; leedh830@naver.com; 2 Department of Emergency Medicine, College of Medicine, Yonsei University, Seoul 03722, Republic of Korea; 3 Division of Cardiology, Severance Cardiovascular Hospital, Integrative Cardiovascular Imaging Center, College of Medicine, Yonsei University, Seoul 03722, Republic of Korea; hjchang@yuhs.ac

**Keywords:** emergency medical services (EMSs), emergency medical dispatch, artificial intelligence, deep learning, machine learning, large language model

## Abstract

**Highlights:**

**What are the main findings?**

- This scoping review found that recent studies have increasingly examined operational decision support, including applications involving multimodal data and large language models.

**What are the implications of the main findings?**

- These findings provide a comprehensive overview of research trends and highlight the need for prospective and real-world evaluation to establish the safety, equity, reliability, and clinical utility of AI-assisted EMS dispatch systems before routine implementation.

**Abstract:**

**Objective:** This study aimed to systematically review worldwide research on artificial intelligence (AI) applications in emergency medical service (EMS) call-taking and dispatch operations and identify current research trends and research needs. **Methods:** This scoping review was reported in accordance with PRISMA-ScR. PubMed, Embase, Scopus, KoreaMed, IEEE Xplore, and the ACM Digital Library were searched for studies published from January 2021 to August 2026. Data were synthesized descriptively. **Results:** Eighteen studies from 13 countries and one multinational setting were included; eight (44.4%) were conducted in Europe. Patient Recognition and Classification was the predominant domain (11/18, 61.1%), followed by Dispatch Support and Operational Decision-Making (5/18, 27.8%) and Other Methodological Applications (2/18, 11.1%). Structured clinical or dispatch data were used in 11 studies, textual data in eight, and call audio in five; four applied multimodal integration. Deep learning (DL) was used as a primary model in 11 studies and classical machine learning (ML) in eight, including three using both; large language models (LLMs) were used in two. Eleven studies involved retrospective model development and internal validation, whereas only two were randomized controlled trials. None of the 11 model-development studies conducted independent external validation or calibration assessment, and the two randomized trials, one with high risk of bias and the other with some concerns, showed no consistent improvement across dispatch outcomes. **Conclusions:** AI applications span diverse clinical and operational tasks, with multimodal integration, speech technologies, and LLMs broadening their potential scope. Evidence remains dominated by retrospective model development and internal validation; prospective and real-world implementation studies are needed to establish safety and clinical utility before integration into EMS systems.

## 1. Introduction

The demand for emergency medical services (EMSs) has continued to increase worldwide, making emergency call-taking and dispatch increasingly important in terms of ensuring appropriate resource allocation and improving patient outcomes. During this stage, dispatchers assess the patient’s condition based on information provided by callers, determine dispatch priorities, allocate emergency medical resources, and provide pre-arrival instructions [1,2]. However, when the collected information is ambiguous or insufficient, accurately recognizing the patient’s condition can be difficult, potentially resulting in over- or under-triage. Consequently, dispatch decisions remain dependent on the experience and expertise of dispatchers [3]. This global trend is also evident in South Korea. According to the 2025 Statistical Yearbook of 119 EMS, the number of patients transported by the Korean 119 EMS system increased by 9.7% over the past decade [4]. In addition, the number of patients classified as Korean Triage and Acuity Scale (KTAS) Levels 1–3—representing resuscitation, emergent, and urgent cases, respectively, within the five-level triage system—has also increased [5], further emphasizing the importance of the prehospital EMS systems.

Recently, artificial intelligence (AI) has been increasingly used as a clinical decision-support technology across healthcare and its application has expanded to the EMS system. In particular, various studies have applied AI to support early recognition of patient conditions [6], severity classification [7], chest-pain prioritization [8], and dispatcher decision support during EMS call-taking and dispatch [9]. Recent advances in generative AI, large language models (LLMs), and multimodal AI have expanded the potential of AI beyond conventional prediction models toward capturing statistical and contextual relationships in language and generating context-sensitive outputs for decision support. As these technologies continue to evolve, they are expected to support more comprehensive clinical decision-making and facilitate the integration of AI into routine EMS dispatch workflows. Furthermore, as advanced technologies continue to evolve, recent studies emphasize the integration of affective elements and interactive technologies—such as artificial empathy—into clinical and healthcare environments to enhance interpersonal communication and patient well-being [10]. However, most studies to date have focused on specific emergency conditions or on evaluating the performance of individual AI technologies [6,7,8,9], resulting in a fragmented research landscape across diverse topics and stages that makes it difficult to discern overarching trends. Although a previous scoping review examined AI applications across the broader prehospital emergency care continuum up to 2021 [11], a comprehensive synthesis specifically focusing on AI applications in EMS call-taking and dispatch, including recent advances in generative AI, LLMs, and multimodal AI, remains limited. Therefore, a systematic summary of the existing literature is needed to identify the current status and research characteristics of AI applications and to suggest future research directions.

This study aims to systematically examine the literature on AI applications in EMS call-taking and dispatch through a scoping review, summarize research trends and the current status of AI implementation, and provide future research directions based on the current evidence and research landscape.

## 2. Materials and Methods

### 2.1. Study Design

This scoping review examined the current status and research trends of AI applications in EMS call-taking and dispatch and was reported in accordance with the PRISMA-ScR (Preferred Reporting Items for Systematic Reviews and Meta-Analyses Extension for Scoping Reviews) guidelines [12]. To focus on contemporary AI applications relevant to current EMS systems, studies published between January 2021 and August 2026 were considered. PubMed, Embase, Scopus, KoreaMed, Institute of Electrical and Electronics Engineers (IEEE) Xplore, and the Association for Computing Machinery (ACM) Digital Library were searched, with the final search conducted on 8 August 2026. The search strategies were reviewed by the authors and a medical librarian for consistency and reproducibility.

### 2.2. Selection of Studies

A literature search was conducted using combinations of keywords related to EMS call-taking, dispatch operations and AI. The complete search strategy is presented in Table 1. A total of 692 records were identified, including 101 from PubMed, 138 from Embase, 192 from Scopus, 9 from KoreaMed, 86 from IEEE Xplore, and 166 from the ACM Digital Library. Duplicate records were removed using Zotero reference management software (version 9.0.6), resulting in 527 records for title and abstract screening. The eligibility criteria were predefined before title and abstract screening and were applied consistently throughout the study-selection process. No additional backward citation searching, forward citation searching, or manual screening of the reference lists of previous review articles was performed.

Two reviewers independently screened the titles and abstracts according to the predefined eligibility criteria. Studies were eligible if they (1) were original, full-text research articles published between January 2021 and August 2026; (2) examined EMS call-taking or dispatch operations; and (3) developed, evaluated, or applied an AI-based method directly related to call-taking or dispatch operations. No language restrictions were applied at the eligibility stage. However, the database searches were conducted using English search terms only, which may have limited the retrieval of studies indexed only in other languages. Studies published in languages other than English were considered eligible if they were identified through the database search and met the predefined eligibility criteria. For studies published in languages other than English, English translations (when available) or translation tools were used to assess eligibility and extract relevant study information. Original empirical studies were eligible regardless of study design or data source, provided that they met the other eligibility criteria. To apply consistent eligibility criteria across publication types, only full-length, peer-reviewed original research articles were included; therefore, review articles, scoping reviews, perspectives, editorials, commentaries, research letters, conference proceedings, book chapters, and preprints were excluded. Disagreements regarding study eligibility were resolved through discussion and consensus between the two reviewers. Following title and abstract screening, 39 reports were selected for full-text assessment.

Of the 39 reports assessed for eligibility, 21 were excluded; 7 were not directly related to EMS call-taking or dispatch operations, and 14 did not use an AI-based method (Table S1). Finally, 18 studies were included in the review (Figure 1).

ACM, Association for Computing Machinery; AI, artificial intelligence; EMS, emergency medical services; IEEE, Institute of Electrical and Electronics Engineers; PRISMA-ScR, Preferred Reporting Items for Systematic Reviews and Meta-Analyses Extension for Scoping Reviews.

### 2.3. Study Analysis

Data charting was conducted using a structured form based on predefined items. However, no separate formal methodological framework was prospectively adopted, and no review protocol was developed or registered. The charted data included publication year, country (defined as where each study was conducted), study design, study focus, input data, AI methods, validation approach, model performance, and major findings. The two reviewers jointly reviewed the extracted data for consistency and resolved any discrepancies through discussion and consensus. When information in the original article was unclear or insufficiently reported, the reviewers discussed the available evidence, and items that could not be determined were recorded as “unclear” or “NR,” as appropriate. Unless otherwise specified, the total number of included studies was used as the denominator for study-level summaries.

For the descriptive synthesis, the study focus of the included studies was classified into two primary application domains according to the intended application of AI: (1) Patient Recognition and Classification and (2) Dispatch Support and Operational Decision-Making. Studies were assigned to domains according to their primary objective and the direct output of the AI system. Studies predicting patient conditions, disease types, severity, or prognosis were classified as ‘Patient Recognition and Classification’, even when their predictions could subsequently influence dispatch decisions. Studies directly supporting dispatch prioritization, resource allocation, deployment, routing, or other operational decisions were classified as ‘Dispatch Support and Operational Decision-Making’. Studies that did not clearly fit either domain were classified as ‘Other Methodological Applications’ (Figure 2).

AI-related technical characteristics were organized into two components: Data processing and Primary AI model. Data processing described how input data were transformed, represented, or integrated using approaches such as structured data processing, automatic speech recognition (ASR), natural language processing (NLP), speaker diarization, geospatial or spatiotemporal data processing, and multimodal data integration; specific AI models used for these processes were also recorded when reported. Primary AI model referred to the models used for the main classification, prediction, optimization, or generation task. AI models were classified as classical machine learning (Classical ML), comprising non-deep-learning algorithms, or deep learning (DL), comprising multilayer neural-network-based methods; LLMs were classified as a subtype of DL. Data-processing methods and primary AI models were analyzed separately. When the same model contributed to both data processing and the primary analytical task, it was recorded in both components according to its respective role. When a study used multiple methods, each distinct method was recorded; however, each broader category was counted only once per study within each component.

Model performance and relevant evaluation outcomes were extracted, together with the validation methods used in each study. For consistent classification, external validation was defined as the evaluation of a developed model using data independent of model development or selection, including data from a different institution, geographic population, or independently collected dataset. The design of each included study was identified, and methodological quality or risk of bias was assessed using design-specific quality-assessment tools, as applicable. RoB 2 was used for randomized trials [13], QUADAS-3 (Quality Assessment of Diagnostic Accuracy Studies-3) for diagnostic-accuracy studies [14], PROBAST (Prediction model Risk Of Bias Assessment Tool)+AI for prediction-model studies [15], and an adapted QAREL (Quality Appraisal of Reliability Studies) framework for relevant speech- or audio-based studies [16]. Methodological quality or risk of bias was assessed by one reviewer using the applicable design-specific quality-assessment tools. For studies for which no applicable validated appraisal instrument was available, methodological quality was not formally assessed, and these studies were identified as not formally assessed in the Supplementary Materials. 

### 2.4. Declaration of Generative AI-Assisted Technologies

During the preparation of this study, ChatGPT (OpenAI, GPT-5.5) was used solely to suggest synonyms, alternative search terms, and keyword combinations to support the development and refinement of the literature search strategy and to improve the clarity and readability of the manuscript. All suggestions provided by ChatGPT were independently reviewed and verified by the authors, and the final search terms and search strings were determined and finalized by the authors before the database searches were conducted. Gemini 1.5 Pro (Google LLC, Mountain View, CA, USA) was used solely to assist in creating the graphical figures (Figure 2, Figure 3 and Figure 4). Neither AI tool was used to conduct database searches, screen or select studies, extract or analyze data, interpret findings, or make scientific decisions. The authors take full responsibility for the final content of the manuscript.

## 3. Results

A total of 18 studies were included in this review (Table 2). The studies represented 13 individual countries and one multinational research setting, with eight studies (44.4%) conducted in Europe (Figure 3). Sweden [8,17,18] was the most frequently represented country, contributing three studies (16.7%), followed by Denmark [6,19] and Spain [7,20], with two studies each (11.1%). The remaining 11 individual countries each contributed one study (5.6%), while the multinational study [21] included research institutions across China, Hong Kong, Macao, and the United States.

### 3.1. AI Application Purposes

Among the 18 included studies, Patient Recognition and Classification was the predominant domain (11/18, 61.1%), followed by Dispatch Support and Operational Decision-Making (5/18, 27.8%). Two studies (2/18, 11.1%) did not fit either domain and were classified as Other Methodological Applications. The annual number of publications was highest in 2021 (*n* = 6), followed by four studies in 2025, three each in 2022 and 2023, two in 2026, and none in 2024 (Figure 4). Twelve studies (66.7%) were published during the first half of the study period (2021–2023), compared with six (33.3%) during the latter half (2024–2026). Most Patient Recognition and Classification studies were published during 2021–2023 (10/11, 90.9%), whereas most Dispatch Support and Operational Decision-Making studies (3/5, 60.0%) and all studies classified as Other Methodological Applications (2/2, 100%) were published during 2024–2026.

#### 3.1.1. Patient Recognition and Classification

Eleven studies were classified as Patient Recognition and Classification. Ten were retrospective studies, including conventional model-development and internal-validation studies, temporal-validation studies, and a post-implementation error analysis, while one was a prospective randomized controlled trial [6]. Out-of-hospital cardiac arrest (OHCA) was the most frequently investigated specific condition, with three studies focusing on its detection during emergency calls [6,17,19]. One study predicted multiple time-critical conditions, including sepsis, myocardial infarction, and cardiac arrest [22]. Two studies assessed the severity of road-traffic injuries [23,25], while two multimodal studies predicted life-threatening status, response delay, and emergency system jurisdiction [7,20]. The remaining studies addressed risk classification in patients with chest pain [8], three-level acuity classification [26], and classification of emergency calls into four complaint categories [24].

#### 3.1.2. Dispatch Support and Operational Decision-Making

Five studies were classified as Dispatch Support and Operational Decision-Making. Three involved retrospective model development and validation [27,28,29], one was a scenario-based LLM–expert agreement study [30], and one was a prospective randomized controlled trial [18]. Three studies focused on dispatch prioritization, including prediction of the need for a lights-and-sirens response to traffic crashes, selection of the highest-priority ambulance request, and risk-based prioritization of low-acuity patients during resource constraints [18,27,30]. One study predicted ambulance response time to support AED-delivery drone dispatch [28], and another optimized ambulance routes using patient, location, and real-time traffic data [29].

#### 3.1.3. Other Methodological Applications

Two simulation-based studies that did not directly focus on patient recognition or operational dispatch decisions were classified as Other Methodological Applications. One developed and evaluated an LLM-based multi-agent system for simulating emergency medical dispatcher–caller interactions [21]. The other constructed a simulated French emergency dispatch dialogue dataset and evaluated baseline models for speaker diarization, ASR, and NLP [31].

### 3.2. Input Data Modalities and Data-Processing Methods

The most frequently used input data were structured clinical or dispatch datasets, which were used in 11 studies [7,8,18,20,21,25,26,27,28,29,30]. Textual input data, including emergency-call transcripts, dispatcher-generated text, and electronic health record (EHR)-derived text, were used in eight studies [7,18,20,21,22,23,27,31]. Call audio data were used in five studies [6,17,19,24,31], primarily to analyze callers’ speech or support automated processing of emergency calls. Four studies integrated structured clinical or dispatch data with dispatcher-generated text within their analytical pipelines [7,18,20,27]. In addition, two studies expanded their training datasets using data augmentation [22,28]. Consistent with the input modalities described above, NLP was the most frequently used data-processing method, appearing in 13 studies (72.2%) [6,7,17,18,19,20,21,22,23,24,27,30,31]. ASR was applied to call audio in five studies (27.8%) [6,17,19,24,31], and speaker diarization was additionally reported in one study (5.6%) [31]. Multimodal integration of structured and textual data was identified in four studies (22.2%) [7,18,20,27]. Geospatial data processing was used in one study of drone dispatch (5.6%) [28], while spatiotemporal data processing was applied in one study of real-time ambulance route optimization (5.6%) [29]. Three studies used structured data without reporting a specialized data-processing method [8,25,26]. Because individual studies could apply more than one data-processing method, these categories were not mutually exclusive.

### 3.3. Primary AI Models

Among the primary AI models, DL was used in 11 studies (61.1%) [6,7,17,19,20,21,23,25,28,29,30], while Classical ML was used in eight studies (44.4%) [8,18,22,23,26,27,28,29]. Three studies evaluated both Classical ML and DL models [23,28,29]. LLMs, classified as a subtype of DL, were used as the primary AI model in two studies (11.1%) [21,30]. The primary model category was not reported in one study (5.6%) [24], while no primary AI model was applicable to one dataset-development study (5.6%) [31]. Because some studies used models from more than one category, these categories were not mutually exclusive.

Within the Patient Recognition and Classification domain (*n* = 11), DL was used in seven studies (63.6%) [6,7,17,19,20,23,25], and Classical ML was used in four studies (36.4%) [8,22,23,26]. One study did not report the model category used for its primary NLU classification task (9.1%) [24], and no study used an LLM as its primary model. Within the Dispatch Support and Operational Decision-Making domain (*n* = 5), Classical ML was used in four studies (80.0%) [18,27,28,29], while DL was used in three studies (60.0%) [28,29,30]. One study used an LLM as its primary model (20.0%) [30]. In the Other Methodological Applications domain (*n* = 2), one study used an LLM-based DL model (50.0%) [21], while the dataset-development study did not have an applicable primary AI model (50.0%) [31].

### 3.4. Performance Evaluation of AI Models

The 18 included studies were classified into four methodological categories: prospective randomized controlled trials (*n* = 2), retrospective evaluations of existing models (*n* = 2), retrospective model-development and internal-validation studies (*n* = 11), and scenario- or simulation-based evaluations (*n* = 3) (Table S2). Because these measures assess different performance constructs, including sensitivity, specificity, F1 score, accuracy, WER, agreement coefficients, expert judgments, and operational outcomes, their values were not directly comparable across studies, even when reported within the same study-design category.

Two prospective randomized controlled trials evaluated the clinical impact of ML-assisted dispatch. Blomberg et al. [6] reported that the ML alert had higher sensitivity than usual dispatch for OHCA detection (85.0% vs. 77.5%) but lower specificity (97.4% vs. 99.6%) and positive predictive value (PPV) (17.8% vs. 55.8%; all *p* < 0.001). However, ML assistance did not significantly improve overall dispatcher recognition (93.1% vs. 90.5%, *p* = 0.15), recognition time (1.71 vs. 1.70 min, *p* = 0.90), or CPR (cardiopulmonary resuscitation) instruction delivery (64.8% vs. 61.9%, *p* = 0.47). Spangler et al. [18] found that ML-assisted risk assessment was associated with modestly higher correct prioritization during resource-constrained situations (68.3% vs. 62.5%; OR 1.28, 95% CI 1.00–1.63; *p* = 0.047) and composite-outcome prioritization (69.6% vs. 63.0%; OR 1.31, 95% CI 1.01–1.72; *p* = 0.041). Overall, the randomized evidence indicated modest operational benefits, without a clear improvement across all dispatch outcomes.

Two studies retrospectively evaluated an existing ML-based OHCA detection system following real-world implementation. Byrsell et al. [17] assessed real-time OHCA recognition and reported 86% sensitivity, with earlier recognition by ML than by dispatchers (median, 72 vs. 94 s). Blomberg et al. [19] examined prediction errors after implementation and reported 84.5% sensitivity, 97.1% specificity, 17.1% PPV, and 99.9% NPV; false negatives were commonly associated with misleading symptom descriptions, reports of normal breathing, and language barriers.

Eleven studies involved retrospective model development and internal validation. Costa et al. [24] separately evaluated the speech-processing component, reporting a WER (word error rate) of 42.12% for Wav2Vec 2.0, compared with 59.96% for HuBERT (Hidden-Unit Bidirectional Encoder Representations from Transformers) and 55.18% for WavLM; the downstream complaint classifier achieved an F1 score of 0.745. Among studies using existing dispatch practice or protocols as comparators, Ceklic et al. [27] reported an F1 score of 0.960 versus 0.228, while Ferri et al. (2021) [7] reported macro F1 scores of 0.759, 0.576, and 0.757 versus 0.634, 0.401, and 0.706 for life-threatening events, response delay, and jurisdiction, respectively. Ferri et al. (2025) [20] reported corresponding F1 scores that were higher by 18.46, 25.96, and 3.63 percentage points. Jamal Chu et al. [28] estimated that their model could reduce median response time from 5.8 to 3.9 min while reducing drone flights by up to 30%. Findings were mixed in the remaining comparative studies: Chin et al. [23] reported that their model achieved 75.0% accuracy versus 76.8% among dispatchers; Wang et al. [26] reported higher accuracy (60.8% vs. 47.6%) and lower over-triage (31.1% vs. 45.7%) but higher under-triage (8.1% vs. 6.7%) than call-center specialists; and Wibring et al. [8] reported different sensitivity–specificity trade-offs for high- and low-risk prediction. Anthony et al. [22] reported 95% accuracy in a 20-case test set, Salah Taamneh et al. [25] reported 65.1% accuracy without a comparator, and Selvan et al. [29] reported 92.3% accuracy for route optimization, although the outcome definition was unclear. None of the 11 studies reported independent external validation or calibration assessment.

Three studies evaluated AI using simulated calls or scenarios rather than patient-level outcomes. Shekhar et al. [30] reported 76.5% agreement between GPT-4o Mini and paramedics, increasing to 93.8% for unanimous panel decisions. In the simulated-call dataset, Nun et al. [31] reported a WER of 16.1% after in-domain fine-tuning, compared with 24.8% for the baseline CTC-wav2vec model and 35.8% for Whisper. Li et al. [21] reported substantial physician agreement (Gwet’s AC1 > 0.70), with correct external-agent contact in 94%, callback instructions in 97%, and appropriate advice in 91% of simulated cases. Although promising, these findings do not establish effectiveness in real-world emergency dispatch.

Quality assessment results are presented in Table S3. Among the randomized controlled trials assessed using Cochrane RoB 2, Blomberg et al. (2021) [6] had some concerns, whereas Spangler et al. [18] had a high overall risk of bias. Both diagnostic accuracy studies, Byrsell et al. [17] and Blomberg et al. (2023) [19], had a high overall risk of bias according to QUADAS-3. All 11 prediction-model studies—Anthony et al. [22], Ferri et al. (2021) [7], Salah Taamneh et al. [25], Chin et al. [23], Wibring et al. [8], Costa et al. [24], Wang et al. [26], Ferri et al. (2025) [20], Jamal Chu et al. [28], Ceklic et al. [27], and Selvan et al. [29]—had a high overall risk of bias according to PROBAST +AI. The agreement study by Shekhar et al. [30], assessed using an adapted QAREL framework, had high methodological and applicability concerns. Nun et al. [31] and Li et al. [21] were not formally assessed because their study designs were not adequately covered by the selected tools.

## 4. Discussion

Most of the included studies were conducted in European countries, accounting for eight of the 18 included studies (44.4%). This geographical concentration may partly reflect the well-established data infrastructure supporting AI research in the region. Several European countries, including Denmark and Sweden, operate nationwide EMS systems with comprehensive digital EMS databases, enabling the collection of large-scale, standardized prehospital data. In addition, countries such as France and Spain have established dedicated prehospital EMS registries, including Service d’Aide Médicale Urgente (SI-SAMU) and the Out-of-Hospital Sudden Cardiac Arrest Registry (OSHCAR), which provide high-quality datasets for AI model development and validation [32,33,34,35]. These infrastructures facilitate the availability of large, interoperable, and routinely collected datasets, which are essential for training and evaluating AI algorithms in the prehospital setting. However, the observed geographical concentration may also have been influenced by the databases searched, language-related eligibility or indexing effects, regional publication practices, data accessibility, and established research networks.

Patient Recognition and Classification was the predominant research focus among the included studies, indicating that AI research in EMS call-taking has primarily focused on identifying, classifying, and predicting patient conditions from information obtained during emergency calls. Among the studies published between 2021 and 2026, a descriptive pattern was observed in which studies published in the later part of the review period also addressed Dispatch Support and Operational Decision-Making in addition to Patient Recognition and Classification. This observation indicates that, within the literature reviewed during this period, AI applications addressed both patient-level prediction and dispatch and operational decision-making. Nevertheless, because these applications directly influence patient safety and EMS resource allocation, algorithm transparency, explainability, and accountability should be considered in addition to predictive performance in the development and implementation of AI systems [36]. Rigorous validation in real-world operational settings is also necessary to establish their clinical and operational utility.

Although some studies used only structured clinical or dispatch data, four studies integrated structured data with dispatcher-generated text. Because emergency medical dispatch is fundamentally a voice-based process, relying solely on structured datasets has inherent limitations in capturing the dynamic interactions between callers and dispatchers. This shift reflects the growing recognition that call audio and dispatcher-generated text provide rich contextual information, including patients’ symptom descriptions, communication patterns, and scene conditions, which cannot be fully represented by structured variables alone. Further advances in multimodal integration may enable structured patient information, call audio, dispatcher narratives, EHR data, wearable-device data, geospatial information, and real-time environmental data to be analyzed together through feature-level or decision-level fusion. These approaches may support more comprehensive patient assessment and dispatch decision-making [37]. However, evidence directly comparing multimodal and single-modality approaches remains limited, highlighting the need for further research to determine the benefits of multimodal integration.

AI applications in EMS call-taking have expanded the scope of research from the analysis of structured patient and dispatch data to the real-time processing of call audio and free-text data through ASR and NLP. Classical ML models have been applied to patient classification and dispatch prioritization and are computationally efficient and applicable to smaller datasets. However, they often depend on manually designed features and have limited capacity to represent complex speech and textual information. DL models enable automated representation learning from speech signals, facilitating the analysis of complex and unstructured audio information [38]. Transformer-based models, including BERT and CamemBERT, provide context-sensitive language representations and enable transfer learning across NLP tasks. However, accent, dialect, multilingual or distressed speech, background noise, speech impairment, caller age, and caller type should be considered during model development because they may adversely affect the performance of speech- and language-processing models. Multimodal approaches may further support EMS decision-making by integrating complementary structured and unstructured information, although challenges remain regarding data alignment, missing modalities, system complexity, and limited direct evidence of superiority over single-modality approaches. In addition, DL approaches, including transformer-based and multimodal models, generally require greater computational resources and careful evaluation of explainability and generalizability. Future research should also consider advanced approaches to data quality assessment and preprocessing. In particular, although outlier detection was not identified among the included studies, it may represent a potential approach for identifying atypical observations in complex medical datasets. Recent methodological research has demonstrated the potential of adaptive outlier-detection methods for medical datasets with different densities and distributions, suggesting that such approaches could be explored in future EMS AI research to support data quality and model robustness [39].

LLMs are a subtype of transformer-based DL with potential applications in EMS call-taking, including support for clinical reasoning, call summarization, automated documentation, pre-arrival instructions, decision support, multilingual communication, and automated call-taking. Although prompt-based use may provide flexibility without conventional model retraining, reliable implementation may still require prompt optimization, local fine-tuning, retrieval augmentation, domain-specific vocabulary adaptation, and site-level validation [40]. LLMs also introduce important concerns related to hallucination, output reliability, response latency, privacy, and regulatory compliance [40,41]. Reinforcement learning and graph neural networks were not represented among the included studies but may provide future opportunities for dynamic resource allocation and network-based modeling of EMS systems, respectively. Their clinical application would require careful consideration of reward design, safety, data quality, and external validation.

Despite technological advances, the current evidence remains insufficient to support widespread clinical implementation of AI in EMS call-taking. The majority of the included studies (11 of 18) involved retrospective model development and internal validation and reported favorable performance for selected tasks; however, none conducted independent external validation or calibration assessment. Consequently, their performance may not generalize to different populations, dispatch systems, or operational settings, and the reliability of their predicted risks remains uncertain. Moreover, favorable model accuracy or discrimination does not necessarily translate into clinical or operational benefits after implementation. This distinction was evident in the two randomized controlled trials. One trial found no significant improvement in overall OHCA recognition, recognition time, or CPR instruction delivery [6]. The other reported only a modest improvement in risk-based prioritization, but this finding should be interpreted cautiously given the study’s high risk of bias [18]. Therefore, predictive performance should be distinguished from clinical utility and readiness for implementation. Achieving clinically meaningful effects in implementation studies may also require local validation and, where necessary, recalibration to account for differences in language, dispatch protocols, disease prevalence, documentation practices, and resource availability across EMS systems. Even after successful clinical implementation, model performance may deteriorate over time as dispatch terminology, protocols, case mix, and resource availability change. AI systems used in practice therefore require ongoing monitoring for temporal dataset shift, periodic recalibration, version control, and post-deployment surveillance to identify performance degradation and emerging safety risks.

Beyond predictive performance and clinical effectiveness, successful implementation requires human-centered AI systems that support dispatcher decision-making and integrate appropriately into existing workflows. Explainable AI methods, such as SHAP (SHapley Additive exPlanations), LIME (Local Interpretable Model-Agnostic Explanations), and attention visualization, may support dispatcher trust and accountability. Complementary uncertainty quantification may further support the safety and reliability of model-guided decisions [42,43]. Such trust and accountability also depend on selecting clinically meaningful performance metrics and acceptable decision thresholds that reflect the potential clinical consequences of model-guided decisions. For OHCA recognition, high sensitivity is essential because false-negative predictions may delay life-saving interventions, whereas specificity and PPV remain important to minimize false-alert burden and alert fatigue. For low-acuity prioritization or ambulance-demand prediction, both under-triage and over-triage may compromise patient safety or inefficiently consume limited EMS resources. Clinically acceptable performance thresholds, including an appropriate balance between sensitivity and specificity, should therefore be established for each dispatch task. However, even when a model demonstrates acceptable reliability, incorrect predictions remain possible. Adequate dispatcher training and safeguards against automation bias are therefore needed to support safe use without promoting excessive reliance on AI recommendations [44,45]. Additionally, enhancing real-world usability requires user-centered systems that integrate seamlessly with existing computer-aided dispatch systems and workflows without imposing additional burdens or causing inconvenience to dispatchers. Furthermore, AI systems should not introduce response latency that could delay decision-making, particularly in time-critical emergency cases. Ensuring timely and reliable operation requires adequate computational infrastructure and sustainable system maintenance.

Despite recent advances, AI remains far from routine implementation in real-world EMS practice. Future research should prioritize prospective multicenter validation and randomized implementation studies to establish the generalizability of AI models across diverse populations and EMS settings. To determine whether improvements in model performance translate into tangible benefits for patients and EMS operations, future studies should evaluate clinically and operationally meaningful outcomes, including recognition time, dispatch delay, response interval, over- and under-triage, dispatcher workload, resource utilization, adverse events, survival, and neurologic outcomes. Current research also remains limited in its integration of heterogeneous data and adaptation to dynamic operational environments. Although these approaches were not identified among the included studies, federated learning, edge AI, EMS digital twins, reinforcement learning, and multimodal foundation models may represent potential areas for future research to address these limitations. Human–AI collaboration and continuous learning frameworks may also support safe implementation and adaptation to changing EMS conditions.

The clinical implementation of AI raises important legal and ethical issues. Dispatchers should retain final decision-making and override authority, with established fallback procedures for low-confidence predictions, incomplete audio, system interruptions, or disagreement with the model. However, because AI recommendations may influence dispatcher decisions, clear accountability frameworks are needed, and AI outputs, overrides, and final decisions should be documented and auditable. Emergency-call data contain sensitive health, location, patient, caller, and bystander information and should therefore be managed under appropriate legal, ethical, and data-governance frameworks, including requirements for consent or lawful secondary use, de-identification, data retention, third-party access, cybersecurity, and auditability [36,46].

### Limitations

This study has several limitations. No formal scoping-review methodological framework was prospectively adopted, and no review protocol was developed or registered, which may have increased the risk of post hoc methodological decisions. The search was limited to PubMed, Embase, Scopus, KoreaMed, IEEE Xplore, and the ACM Digital Library, without backward or forward citation searching; therefore, relevant gray literature and studies not consistently indexed in these databases may not have been captured. Although no language restrictions were applied, the use of English search terms may have reduced the retrieval of studies indexed only in other languages. Conference proceedings and preprints were excluded to maintain consistent publication eligibility. However, because conference proceedings are an important dissemination route in AI and computer science, their exclusion may have resulted in the omission of relevant emerging technical evidence. The risk-of-bias assessment was introduced during revision in response to peer review and was not part of the original review plan. Two studies (Nun et al. [31] and Li et al. [21]) were not formally assessed because no applicable validated appraisal tool was available for their designs.

Limitations of the included evidence should also be considered. Reporting of demographic characteristics and language coverage was incomplete, limiting assessment of dataset diversity, algorithmic bias, and generalizability. Calibration assessment and decision-curve analysis were not reported in the retrospective model-development studies, limiting evaluation of the reliability and potential clinical utility of predicted risks. Outcome definitions, prevalence, reference standards, comparators, and performance metrics differed substantially, making direct performance comparisons inappropriate. Most prediction-model and diagnostic-accuracy studies had a high risk of bias. Differences in EMS systems, dispatch protocols, and healthcare resources across countries further limit the generalizability of the findings.

## 5. Conclusions

This scoping review identified a heterogeneous body of evidence on AI applications in EMS call-taking and dispatch operations. Multimodal data integration, speech technologies, and large language models have broadened the potential scope of these applications. However, the evidence was dominated by retrospective model development and internal validation, with limited external validation. Further prospective evaluation, real-world implementation studies, and assessments of patient outcomes are needed to establish the safety, equity, reliability, and clinical utility of AI before its integration into EMS systems for dispatcher decision support.

## Figures and Tables

**Figure 1:**
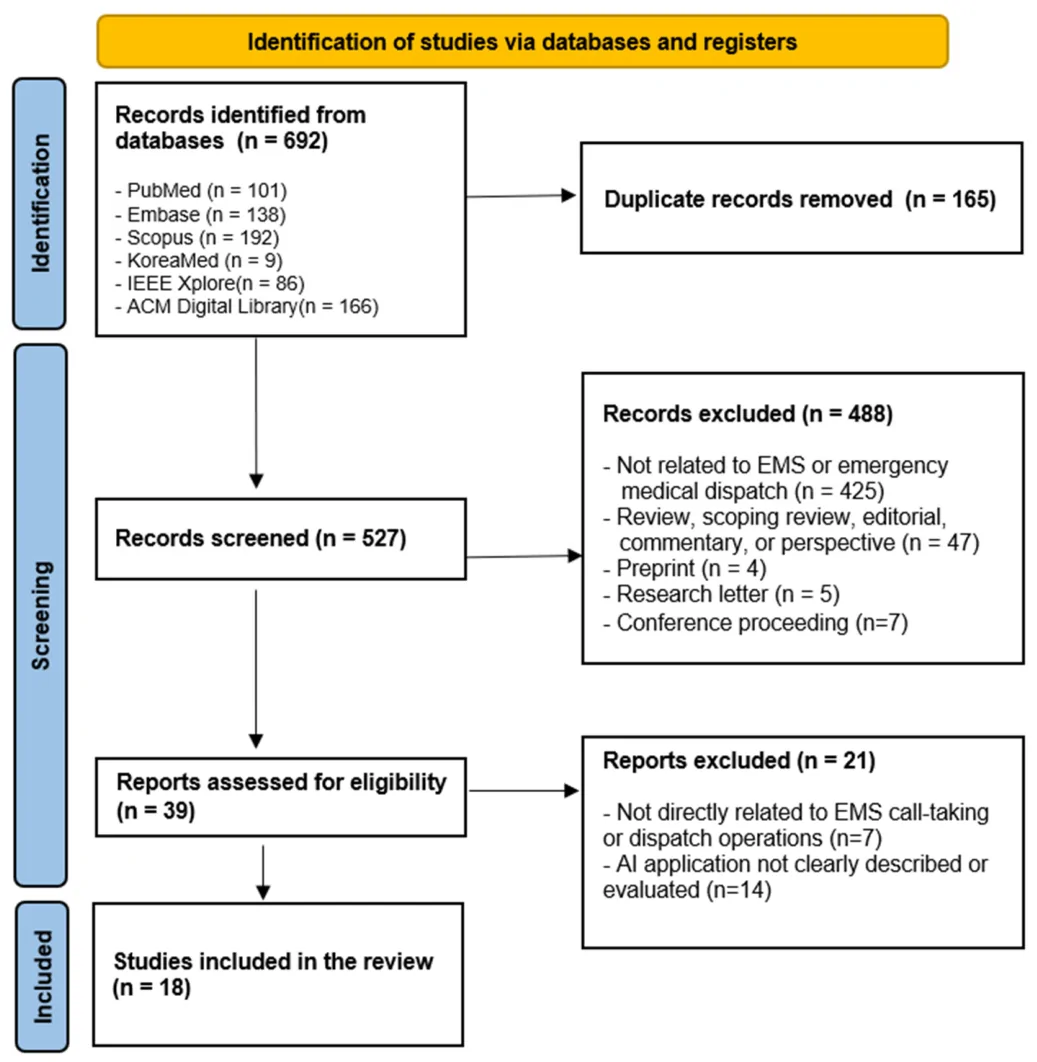
PRISMA-ScR flow diagram.

**Figure 2:**
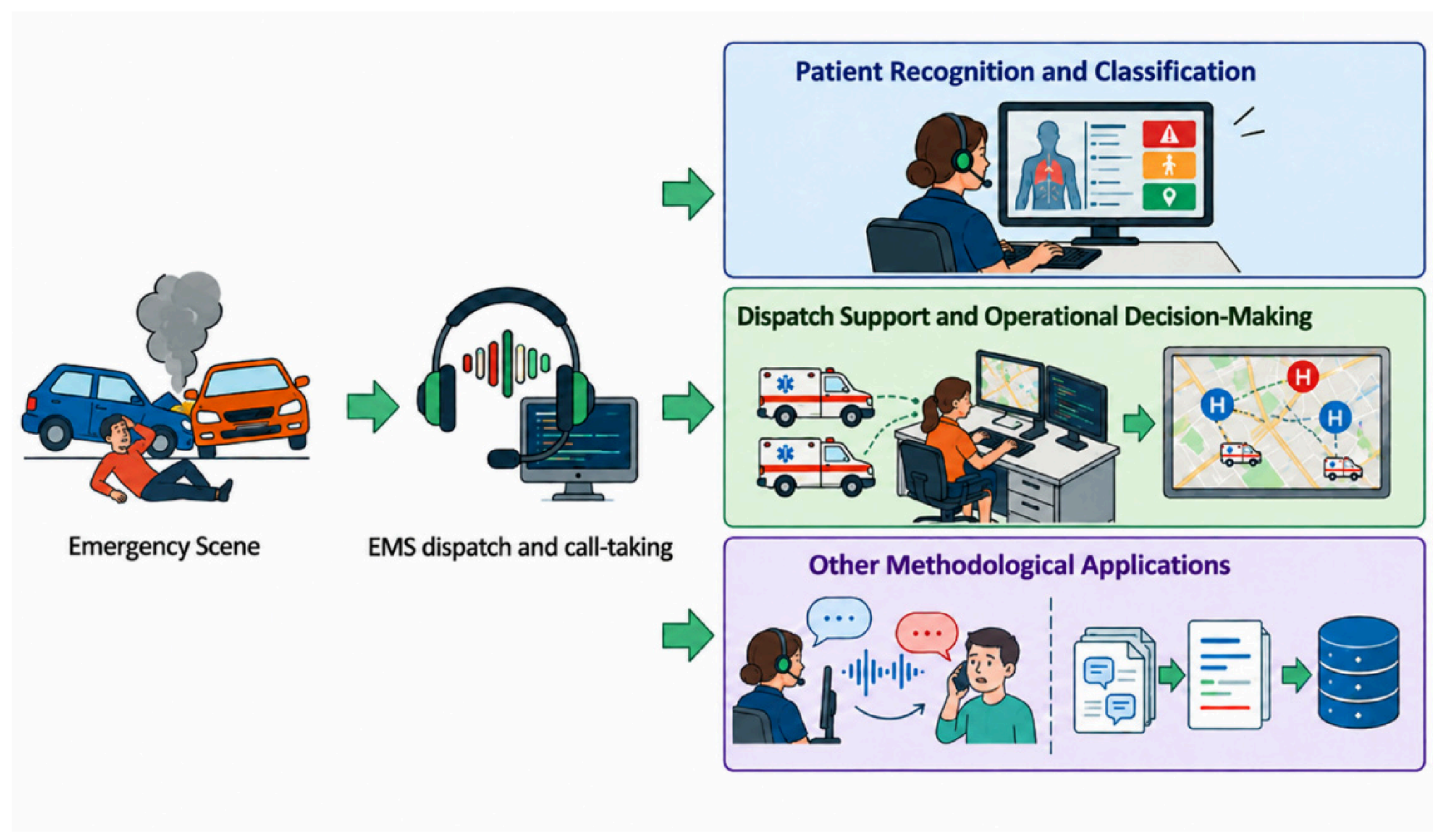
Classification of the included studies according to the research purpose (illustration created using Gemini 1.5 Pro by Google, USA, to assist in visualizing the research methods). EMS, emergency medical service.

**Figure 3:**
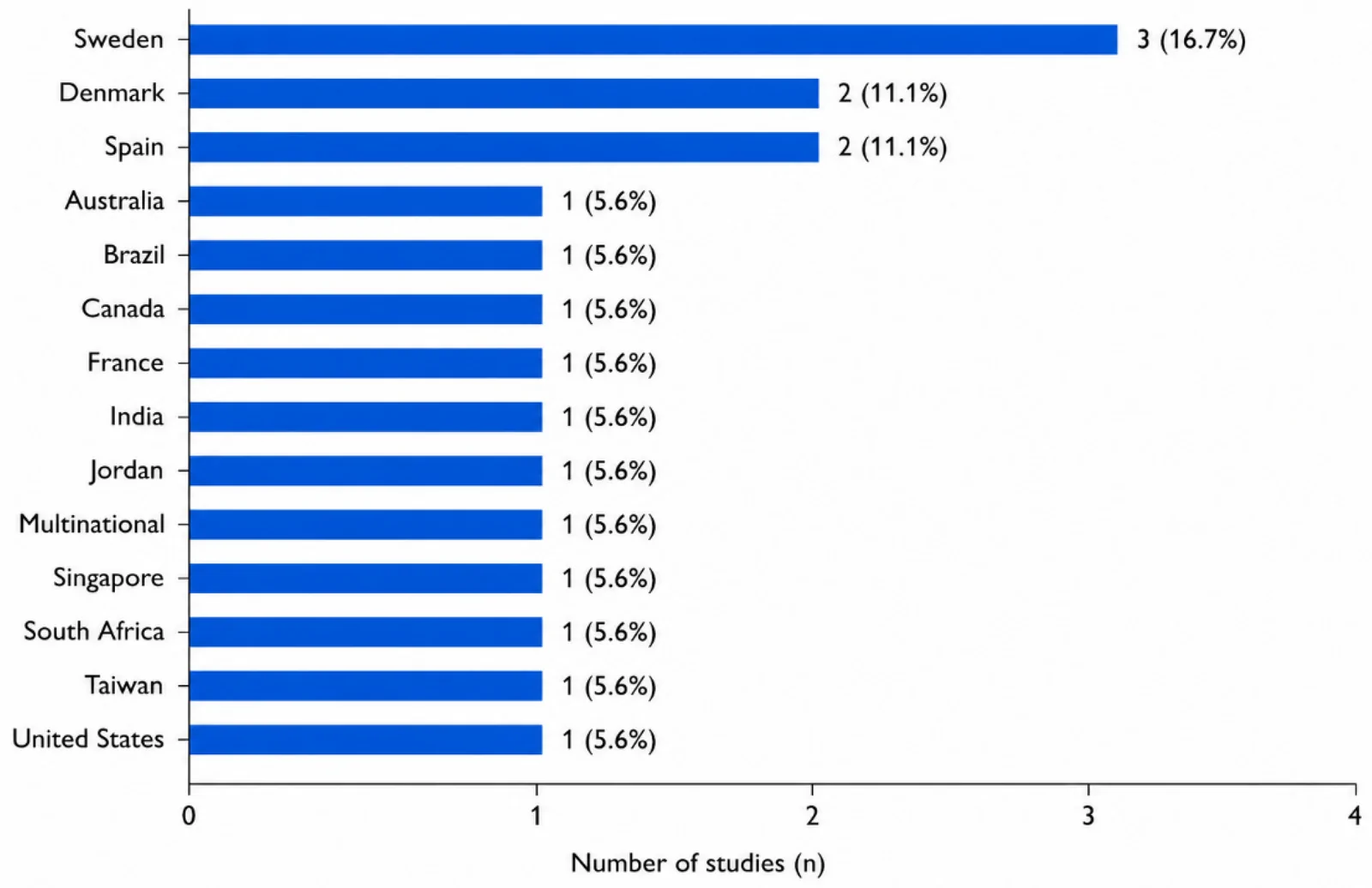
Distribution of included studies by country (illustration created using Gemini 1.5 Pro by Google, USA, to assist in visualizing the research methods).

**Figure 4:**
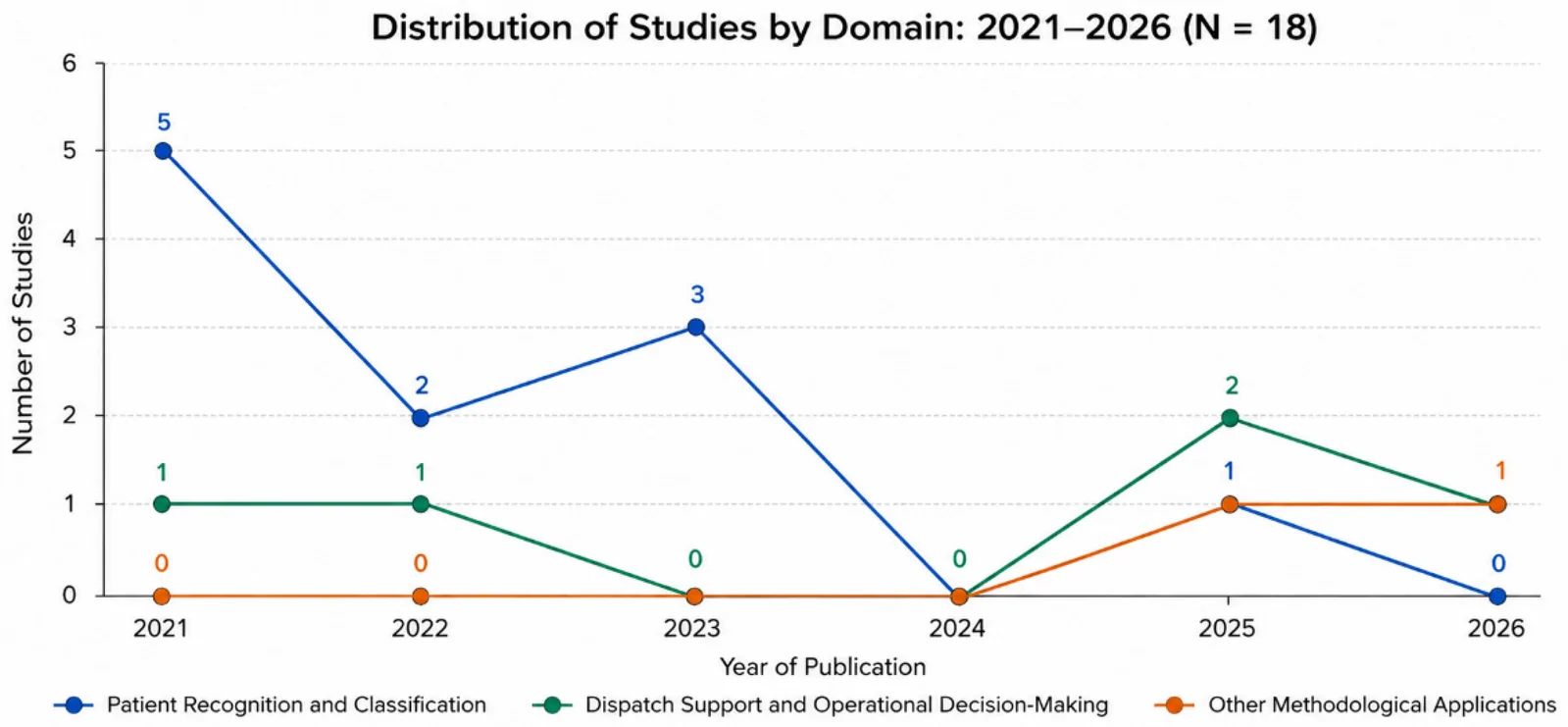
Distribution and publication trends of studies by domain (2021–2026) (Illustration created using Gemini 1.5 Pro by Google, USA, to assist in visualizing the research methods).

**Table 1:** Search strategy.

Database	Search Strategy
PubMed	(“Emergency Medical Services”[Mesh] OR EMS OR prehospital) AND (dispatch* OR dispatcher* OR “medical dispatch*” OR “emergency dispatch*” OR “emergency call*” OR “call-taking*” OR “emergency medical communication centre*” OR “emergency medical communication center*”) AND (“Artificial Intelligence”[Mesh] OR “Artificial Intelligence” OR AI OR “Machine Learning”[Mesh] OR “machine learning” OR “deep learning” OR “large language model*” OR LLM OR “Natural Language Processing”[Mesh] OR “natural language processing” OR NLP OR “automatic speech recognition*” OR ASR)
Embase	(‘emergency medical service’/exp OR EMS OR prehospital) AND (dispatch* OR dispatcher* OR “medical dispatch*” OR “emergency dispatch*” OR “emergency call*” OR “call-taking*” OR “emergency medical communication centre*” OR “emergency medical communication center*”) AND (‘artificial intelligence’/exp OR ‘machine learning’/exp OR ‘natural language processing’/exp OR “Artificial Intelligence” OR AI OR “machine learning” OR “deep learning” OR “large language model*” OR LLM OR “natural language processing” OR NLP OR “automatic speech recognition*” OR ASR)
Scopus	(“Emergency Medical Services” OR EMS OR prehospital) AND (dispatch* OR dispatcher* OR “medical dispatch*” OR “emergency dispatch*” OR “emergency call*” OR “call-taking*” OR “emergency medical communication centre*” OR “emergency medical communication center*”) AND (“Artificial Intelligence” OR “artificial intelligence” OR AI OR “machine learning” OR “deep learning” OR “large language model*” OR LLM OR “natural language processing” OR NLP OR “automatic speech recognition*” OR ASR)
IEEE Xplore	(“Emergency Medical Services” OR EMS OR prehospital) AND (dispatch* OR dispatcher* OR “medical dispatch*” OR “emergency dispatch*” OR “emergency call*” OR “call-taking*” OR “emergency medical communication centre*” OR “emergency medical communication center*”) AND (“Artificial Intelligence” OR “artificial intelligence” OR AI OR “machine learning” OR “deep learning” OR “large language model*” OR LLM OR “natural language processing” OR NLP OR “automatic speech recognition*” OR ASR)
ACM Digital Library	(“Emergency Medical Services” OR EMS OR prehospital) AND (dispatch* OR dispatcher* OR “medical dispatch*” OR “emergency dispatch*” OR “emergency call*” OR “call-taking*” OR “emergency medical communication centre*” OR “emergency medical communication center*”) AND (“Artificial Intelligence” OR “artificial intelligence” OR AI OR “machine learning” OR “deep learning” OR “large language model*” OR LLM OR “natural language processing” OR NLP OR “automatic speech recognition*” OR ASR)
KoreaMed *	EMS AND dispatch AND artificial intelligence; EMS AND dispatcher AND AI;EMS AND emergency call AND machine learning; EMS AND call-taking; EMS AND medical dispatch; EMS AND emergency dispatch; EMS AND prehospital AND deep learning; EMS AND natural language processing; EMS AND automatic speech recognition; EMS AND large language model; 119 AND artificial intelligence; Emergency Medical Services AND dispatch.

* Search syntax was modified according to the characteristics of each database. Final search date: 8 August 2026. ACM, Association for Computing Machinery; AI, artificial intelligence; ASR, automatic speech recognition; EMS, emergency medical services; IEEE, Institute of Electrical and Electronics Engineers; LLM, large language model; MeSH, Medical Subject Headings; NLP, natural language processing.

**Table 2:** Characteristics of the included studies and AI approaches, grouped by application domain.

Domain	Author (Year)	Country	Study Design	Input Data and Language	Data Processing	Primary AI Model	AI Task
(1) Patient Recognition and Classification	Anthony et al. (2021) [22]	South Africa	Retrospective model-development and internal-validation study	Emergency-call transcripts; English, Afrikaans, isiZulu, and Sesotho	NLP: machine translation, TF-IDF, and handcrafted feature extraction	Classical ML: SVM, logistic regression, random forest, and k-NN	Prediction of sepsis, myocardial infarction, and cardiac arrest
	Blomberg et al. (2021) [6]	Denmark	Prospective randomized controlled trial	Emergency-call audio; Danish	ASR: DL model NR; NLP: DL model NR	DL: OHCA detection model (specific method NR)	OHCA detection during emergency calls
	Blomberg et al. (2023) [19]	Denmark	Retrospective post-implementation error-analysis study	Emergency-call audio; Danish	ASR: DL model NR; NLP: DL model NR	DL: OHCA detection model (specific method NR)	OHCA detection during emergency calls
	Byrsell et al. (2021) [17]	Sweden	Retrospective temporal internal-validation study	Emergency-call audio; Swedish	ASR: DL—CTC-based DNN; NLP: DL—densely connected DNN	DL: densely connected DNN	Real-time OHCA detection
	Chin et al. (2022) [23]	Taiwan	Retrospective model-development and internal-validation study	Emergency-call transcripts; Mandarin Chinese	NLP: TF-IDF-based feature extraction	Classical ML: k-NN, decision tree, SVM, multinomial naive Bayes, and Bernoulli naive Bayes; DL: MLP	Severe road-traffic trauma prediction
	Costa et al. (2023) [24]	Brazil	Retrospective model-development and internal-validation study	Emergency-call audio; Brazilian Portuguese	ASR: DL—Wav2Vec 2.0, HuBERT, and WavLM; NLP: NLU classification model NR	NLU classification model: NR	Four-class emergency complaint classification
	Ferri et al. (2021) [7]	Spain	Retrospective model-development and internal-validation study	Structured clinical data; Dispatcher text; Spanish	NLP: DL—BERT-based text representation; multimodal data integration: DL—DeepEMC2	DL: DeepEMC2 comprising MLP, BLSTM, and BERT	Prediction of life-threatening level, response delay, and emergency system jurisdiction
	Ferri et al. (2025) [20]	Spain	Retrospective continual model-development and temporal internal-validation study	Structured clinical data; Dispatcher text; Spanish	NLP: DL—BERT-based text representation; multimodal data integration: DL—CliTexNet	DL: CliTexNet, a BERT-based multimodal DNN	Prediction of life-threatening level, response delay, and emergency system jurisdiction
	Salah Taamneh et al. (2021) [25]	Jordan	Retrospective model-development and internal-validation study	Structured road traffic accident data; Language: NA	Structured data processing	DL: ANN	Road-traffic injury severity prediction
	Wang et al. (2023) [26]	Singapore	Retrospective model-development and internal-validation study	Structured clinical data; Language: NA	Structured data processing	Classical ML: logistic regression, decision tree, XGBoost, and random forest	Three-level acuity classification
	Wibring et al. (2022) [8]	Sweden	Retrospective model-development and internal-validation study	Structured clinical data; Language: NA	Structured data processing	Classical ML: logistic regression	High- and low-risk prediction in patients with chest pain
(2) Dispatch Support and Operational Decision-Making	Ceklic et al. (2022) [27]	Australia	Retrospective model-development and internal-validation study	Structured MPDS code; Dispatcher text; English	NLP: bag-of-words feature extraction; multimodal data integration	Classical ML: ensemble classifier, k-NN, naive Bayes, and SVM	Prediction of the need for a lights-and-sirens response to traffic crashes
	Jamal Chu et al. (2021) [28]	Canada	Retrospective model-development and simulation-based internal-validation study	Structured spatiotemporal EMS dispatch data; Language: NA	Structured data processing; geospatial data processing	Classical ML: linear regression; DL: neural network	Ambulance response time prediction for AED-delivery drone dispatch
	Selvan et al. (2025) [29]	India	Retrospective model-development and internal-validation study	Structured patient, location, and real-time traffic data; Language: NA	Structured data processing; spatiotemporal data processing: DL—CRNN	Classical ML: decision tree and SVM; DL: CRNN	Real-time ambulance route optimization
	Shekhar et al. (2025) [30]	United States	Scenario-based LLM–expert agreement study	Structured ambulance request type; English	NLP: DL—LLM (GPT-4o mini)	DL: LLM—GPT-4o mini	Selection of the highest-priority request type among four options
	Spangler et al. (2026) [18]	Sweden	Prospective randomized controlled trial	Structured clinical data; Dispatcher text; Language: NR	NLP: bag-of-words feature extraction; multimodal data integration	Classical ML: gradient boosting	Risk-based prioritization among low-acuity patients during resource constraints
(3) Other Methodological Applications	Li et al. (2026) [21]	Multinational	Simulation-based multi-agent system development and evaluation study	Structured clinical data; EHR-derived text; English	NLP: DL—LLM (GPT-4o)	DL: LLM—GPT-4o	Simulation of emergency medical dispatcher–caller interactions
	Nun et al. (2025) [31]	France	Simulation-based dataset-development and baseline-model evaluation study	French-language simulated dispatch call audio and transcripts; French	Speaker diarization: DL—ECAPA-TDNN; ASR: DL—Wav2Vec 2.0-CTC and Whisper; NLP: DL—CamemBERT	NA	Emergency medical dispatch dialogue dataset construction

AED, automated external defibrillator; AI, artificial intelligence; ANN, artificial neural network; ASR, automatic speech recognition; BERT, Bidirectional Encoder Representations from Transformers; BLSTM, bidirectional long short-term memory; CRNN, convolutional recurrent neural network; CTC, connectionist temporal classification; DL, deep learning; DNN, deep neural network; ECAPA-TDNN, Emphasized Channel Attention, Propagation and Aggregation in Time Delay Neural Network; EHR, electronic health record; EMS, emergency medical services; GPT, generative pre-trained transformer; HuBERT, Hidden-Unit BERT; k-NN, k-nearest neighbors; LLM, large language model; ML, machine learning; MLP, multilayer perceptron; MPDS, Medical Priority Dispatch System; NA, not applicable; NLP, natural language processing; NLU, natural language understanding; NR, not reported; OHCA, out-of-hospital cardiac arrest; SVM, support vector machine; TF-IDF, term frequency–inverse document frequency; XGBoost, extreme gradient boosting.

## Data Availability

No new primary or patient-level data were generated. All study-level extraction and coding data used for the descriptive synthesis, including domain, data modality, AI-model classification, validation category, and risk-of-bias judgments, are provided in Table 2 and Supplementary Tables S2 and S3.

## Supplementary Materials

The following supporting information can be downloaded at: https://www.mdpi.com/article/10.3390/healthcare14193176/s1, Table S1: Studies excluded after full-text assessment; Table S2: Model performance and main findings of the included studies; Table S3: Quality assessment of the included studies.
