# Supplementary material for: Artificial Intelligence in Emergency Medical Call-Taking and Dispatch Operations: A Scoping Review
Source: Healthcare (Basel). 2026 Sep 24;14(19):3176. doi: 10.3390/healthcare14193176 (PMC13665075; doi:10.3390/healthcare14193176)
Supplement: Supplementary file 1 [file healthcare-14-03176-s001.zip › healthcare-4467440-supplementary.pdf]

## Supplementary Materials

**Table S1. Studies excluded after full-text assessment**

| | Author | Year | Title | Reason for exclusion |
|---|---|---|---|---|
| 1 | Cano et al. | 2026 | Circumstances associated with fatality from opioid overdose at the time of contact with emergency medical services | Excluded because it investigates retrospective logistic regression correlates of opioid overdose fatalities during EMS activations without utilizing artificial intelligence or focusing on emergency call-taking or dispatch operations. |
| 2 | Castro Delgado et al. | 2021 | Potential capacity of an emergency dispatch center to predict COVID-19–related hospital and intensive care unit admissions | Excluded because it investigates the epidemiological correlation between emergency dispatch call volumes and COVID-19 hospital admissions using observational data without utilizing artificial intelligence. |
| 3 | Chen et al. | 2022 | Artificial intelligence-assisted remote detection of ST-elevation myocardial infarction using a mini-12-lead electrocardiogram device in prehospital ambulance care | Excluded because it focuses on prehospital ambulance care and the remote detection of ST-elevation myocardial infarction using a mini-12-lead ECG device rather than emergency medical services call-taking or dispatch operations. |
| 4 | Dos Santos Cabral et al. | 2021 | Metaheuristics in the decentralization of SAMU bases using simulation in northeastern Brazil | Excluded because it used a mathematical model and simulation rather than an artificial intelligence-based method. |
| 5 | Gellert et al. | 2024 | The potential of virtual triage AI to improve early detection, care acuity alignment, and emergent care referral of life-threatening conditions | Excluded because it focuses on an AI-based virtual triage engine used by patient-users rather than emergency medical services call-taking or dispatch operations. |
| 6 | Guigues et al. | 2026 | New heuristics for the operation of an ambulance fleet under uncertainty | Excluded because it does not utilize artificial intelligence or machine learning methods, focusing instead on traditional heuristic and stochastic |

| | | | | |
|---|---|---|---|---|
| | | | | optimization algorithms for ambulance operations. |
| 7 | Isenberg et al. | 2022 | Optimizing Prehospital Stroke Systems of Care-Reacting to Changing Paradigms (OPUS-REACH): a pragmatic registry of large vessel occlusion stroke patients to create evidence-based stroke systems of care and eliminate disparities in access to stroke care | Excluded because it is a pragmatic registry and observational study focusing on prehospital transport and 90-day outcomes of LVO stroke patients, which does not involve the development, evaluation, or application of an AI-based method. |
| 8 | Iversen et al. | 2025 | Stroke Sensitivity Calculation in Medical Emergency Calls and Factors Associated With Stroke Suspicion: A Retrospective Registry-Based Study | Excluded because it explores emergency medical communication center stroke sensitivity and associated factors using traditional descriptive and multivariable logistic regression analyses to lay the foundation for future AI tools, rather than developing, evaluating, or applying an AI-based method. |
| 9 | Johansen et al. | 2026 | Timing is survival: modeling how earlier calls improve cardiac arrest outcomes | Excluded because the study used survival modeling and Monte Carlo simulation rather than an artificial intelligence-based method. |
| 10 | Lee et al. | 2025 | Impact of Emergency Medical Services Call–to–Hospital Arrival Time on Outcomes of Cardiac Arrest: A Nationwide Prospective Cohort | Excluded because the study did not use artificial intelligence, but instead evaluated clinical outcomes based on emergency medical services call-to-hospital arrival times using conventional statistical analyses. |
| 11 | Meyer et al. | 2026 | VISIION-L: Viz.ai implementation of stroke augmented intelligence and communications platform to improve indicators and outcomes for a comprehensive stroke center and network – Life Flight. A pilot experience | Excluded because it focuses on inter-facility patient transfer, aeromedical communications, and multi-team coordination using an AI platform rather than emergency medical services call-taking or dispatch operations. |

| | | | | |
|---|---|---|---|---|
| 12 | Moshe et al. | 2026 | Between Compliance and Complexity: Dispatchers' Use of an Automatic Emergency Vehicle Assignment System | Excluded because the study did not utilize artificial intelligence, machine learning, deep learning, or NLP as a research method, but instead performed statistical regression analyses on operational emergency dispatch records. |
| 13 | Nguyen et al. | 2023 | Prediction of Out-of-Hospital Cardiac Arrest Survival Outcomes Using a Hybrid Agnostic Explanation TabNet Model | Excluded because it focuses on predicting patient survival outcomes (prognosis) rather than operating at the emergency call reception or dispatch stage. |
| 14 | Nicoletta et al. | 2025 | Performance measures of the medical priority dispatch system in an urban basic life support system | Excluded because the study evaluated dispatch prioritization using conventional statistical methods without developing, evaluating, or applying an artificial intelligence-based method for emergency medical services call-taking or dispatch operations. |
| 15 | Pilbery et al. | 2025 | What factors predict ambulance prealerts to the emergency department? Retrospective observational study from three UK ambulance services | Excluded because it is a retrospective observational study analyzing factors predicting ambulance prealerts using Least Absolute Shrinkage and Selection Operator (LASSO) and multivariable logistic regression models, which does not involve the development, evaluation, or application of an AI-based method. |
| 16 | Rautenstrauss et al. | 2023 | Ambulance dispatching during a pandemic: Tradeoffs of categorizing patients and allocating ambulances | Excluded because the study did not use artificial intelligence, but instead constructed a model using mathematical optimization and queuing methods for emergency medical services dispatch. |

| | | | | |
|---|---|---|---|---|
| 17 | Rayborn et al. | 2025 | The Race Against Sepsis: Are Helicopters Always Necessary? A Single-Center Analysis at a Tertiary Pediatric Hospital | Excluded because the study evaluated ground versus rotor-wing transport for pediatric patients with suspected sepsis rather than an AI-based method directly related to EMS call-taking or dispatch operations. |
| 18 | Waalwijk et al. | 2022 | Priority accuracy by dispatch centers and Emergency Medical Services professionals in trauma patients: a cohort study | Excluded because the study did not use artificial intelligence, but instead evaluated the priority-setting accuracy of dispatch centers and emergency medical services professionals using conventional observational and statistical analyses. |
| 19 | Wang et al. | 2022 | Task Offloading for Post-Disaster Rescue in Unmanned Aerial Vehicles Networks | Excluded because it focuses on fog computing-based task offloading and matching algorithms between unmanned aerial vehicles and ground vehicles for post-disaster rescue, which does not examine emergency medical services call-taking or dispatch operations. |
| 20 | Xu et al. | 2024 | The emergency medical service dispatch recommendation system using simulation based on bed availability | Excluded because the study did not use artificial intelligence, but instead constructed a forecasting and hospital recommendation system for emergency medical services dispatch using simulation and routing integration methods. |
| 21 | Zhao et al. | 2025 | Optimizing Helicopter Air Ambulance Dispatch to Improve Sustainability in Interfacility Transfers | Excluded because it focuses on mathematical optimization strategies for helicopter air ambulance dispatch during interfacility transfers to reduce emissions and fuel use, rather than developing, evaluating, or applying an AI-based |

| | | | | |
|---|---|---|---|---|
| | | | | method for EMS emergency medical call-taking or dispatch operations. |

AED, automated external defibrillator; AI, artificial intelligence; COVID-19, coronavirus disease 2019; ECG, electrocardiogram; EMS, emergency medical services; LVO, large vessel occlusion; ML, machine learning; NLP, natural Language Processing; SAMU, Serviço de Atendimento Móvel de Urgência.

**Table S2. Model performance and main findings of the included studies**

| Study design | Author (Year) | AI task | Study/EMS setting | Data-collection period | Sample and dataset characteristics | Reference standard | Intervention vs comparator | Validation status | Implementation status | Model performance | Main results | Conclusion |
|---|---|---|---|---|---|---|---|---|---|---|---|---|
| Prospective randomized controlled trial | Blomberg et al. (2021) [6] | OHCA detection during emergency calls | Copenhagen EMS, Capital Region of Denmark | Sep 2018–Dec 2019 | 2,581 vs 2,661 calls; 318 vs 336 OHCA | OHCA status confirmed through linkage with the Danish Cardiac Arrest Registry | ML-assisted dispatch (OHCA alert) vs usual dispatch | Prospective randomized evaluation | Real-world clinical implementation trial | Sensitivity: 85.0% vs 77.5%; specificity: 97.4% vs 99.6%; PPV: 17.8% vs 55.8% (all $p < 0.001$); Calibration: NR. | OHCA recognition: 93.1% vs 90.5% ($p = 0.15$); recognition time: 1.71 vs 1.70 min ($p = 0.90$); CPR instruction: 64.8% vs 61.9% ($p = 0.47$) | ML showed higher sensitivity but did not significantly improve dispatcher recognition. |
| | Spangler et al. (2026) [29] | Risk-based prioritization among low-acuity patients during resource constraints | Emergency medical dispatch centers in Uppsala and Västmanland, Sweden | Feb 2021–Dec 2024; second site from Jun 2024 | 660 vs 585 RCS; 1,479 vs 1,285 patients | Highest NEWS2 calculated from vital signs subsequently collected by ambulance personnel | ML-assisted dispatch (risk assessment) vs usual dispatch | Prospective randomized evaluation | Real-world clinical implementation trial | C-index: 0.583–0.746; high-confidence accuracy: 78.5%; low-confidence accuracy: 67.4%; sensitivity and specificity: NR; Limited calibration assessment by age, sex, and | Correct prioritization: 68.3% vs 62.5%; OR 1.28 (95% CI 1.00–1.63, $p = 0.047$); composite-outcome prioritization: 69.6% vs 63.0%; OR 1.31 (95% CI 1.01–1.72, $p = 0.041$) | ML modestly improved patient prioritization, but further validation is required. |

| | | | | | | | | | | | | |
|---|---|---|---|---|---|---|---|---|---|---|---|---|
| | | | | | | | | | | complaint category. | | |
| Retrospective evaluation of an existing model | Blomberg et al. (2023) [18] | OHCA detection during emergency calls | Copenhagen EMS, Capital Region of Denmark | Sep 2018–Dec 2019 | 169,049 processed emergency calls; 1,177 confirmed OHCAs. Missing-data imputation: NR. | OHCA status confirmed through linkage with the Danish Cardiac Arrest Registry | Existing ML-based OHCA detection vs. confirmed OHCA status and dispatcher recognition | Post-implementation evaluation | Implemented in routine EMS practice | Sensitivity 84.5%; specificity 97.1%; PPV 17.1%; NPV 99.9%. Calibration: NR. | 995 TP, 182 FN, 4,816 FP, and 163,075 TN. Among audited FNs, 31.3% involved descriptions of other conditions, 27.6% reported normal breathing, and 22.7% involved language barriers. | The model showed high sensitivity and specificity but low PPV. Errors were commonly related to misleading breathing descriptions, language barriers, and unconscious or clearly deceased patients. |
| | Byrsell et al. (2021) [19] | Real-time OHCA detection | SOS Alarm emergency medical dispatch system, Sweden | 2018 | 851 confirmed OHCA calls and 85,205 non-OHCA calls from 2018. Unavailable, | OHCA status recorded in the Swedish Registry of Cardiopulmonary | Existing ML-based OHCA detection vs dispatchers | Retrospective post-implementation evaluation | Implemented in routine EMS practice | At FPR 1.5: sensitivity 86%, median recognition time 72 s, recognition within 60 s 36%, and | ML vs dispatchers: overall recognition 86% vs 84%; recognition within 60 s 36% vs 25%; | The existing ML model recognized OHCA earlier and more frequently |

| | | | | | | | | | | | |
|---|---|---|---|---|---|---|---|---|---|---|---|
| | | | | | incomplete, or unsuitable audio calls were excluded; missing-data imputation: NR. | Resuscitation | | | | within 90 s 51%. False-alert rate in the non-OHCA cohort: 0.8%. PPV, NPV, dispatcher specificity, AUROC, and calibration: NR. Across FPR settings 1.0–3.0, sensitivity increased from 81% to 90%. | within 90 s 51% vs 40%; median recognition time 72 vs 94 s. Among cases recognized by both, ML was 28 s faster (p<0.001). | than dispatchers, but the optimal false-alert threshold requires prospective evaluation. |
| Retrospective model development and internal validation | Anthony et al. (2021) [17] | Prediction of sepsis, myocardial infarction, and cardiac arrest | Multiple EMS contact centers in South Africa | NR | 93 original transcripts covering sepsis, myocardial infarction, and cardiac arrest; training/test split: 80/20, followed by 10-fold cross-validation in the training set. Training data were augmented and balanced using EDA; augmentation was not | Prehospital diagnostic labels obtained from the original condition-specific studies | SVM with TF-IDF and feature selection vs random forest, logistic regression, k-NN, and dummy classifier | Random internal holdout validation with cross-validation | Model development only | Random 80/20 holdout testing; 10-fold cross-validation within the training set; Best model: SVM with TF-IDF and SelectKBest; cross-validation accuracy: 100% ± 0.00; unseen-test accuracy: 95%. Sensitivity, specificity, precision, recall, F1, | Test accuracy with TF-IDF: SVM 90%, random forest 95%, k-NN 95%, logistic regression 75%, dummy 20%. After tuning/feature selection, the best SVM achieved 95% test accuracy and 100% cross-validation accuracy. | ML model achieved 95% accuracy in classifying the three diagnoses, but the extremely small test set, synthetic augmentation, and lack of external validation substantially limit the reliability |

| | | | | | | | | | | | | |
|---|---|---|---|---|---|---|---|---|---|---|---|---|
| | | | | | applied to the 20-case test set. Missing-data handling: NR. | | | | | AUROC, and calibration: NR. | | and generalizability of the findings. |
| | Ceklic et al. (2022) [25] | Prediction of the need for a lights-and-sirens response to traffic crashes | St John Western Australia EMS, Perth metropolitan area, Australia | Jan 2014–Dec 2016 | 11,971 traffic crashes; training/test split: 60/40, stratified by outcome, with 10-fold cross-validation. Missing-data handling and imputation: NR. | Retrospectively determined need for lights-and-sirens response based on hospital admission, urgent transport, or predefined high-acuity indicators in paramedic records | Ensemble model using EMD text and MPDS codes vs other ML models and existing dispatch practice | Random internal holdout validation with cross-validation | Model development only | Stratified random 60/40 holdout testing; 10-fold cross-validation; Ensemble using text and MPDS codes: precision 0.940 (95% CI 0.933–0.947), recall 0.980 (0.976–0.984), F1 0.960 (0.952–0.968). Specificity, AUROC, and calibration: NR. | Ensemble vs existing practice: recall 0.980 vs 1.000, precision 0.940 vs 0.129, and F1 0.960 vs 0.228. Text-only ensemble achieved precision 0.975, recall 0.974, and F1 0.974. | In internal validation, the EMD text-based model outperformed existing dispatch practice, but prospective external validation is required. |

| | | | | | | | | | | | | |
|---|---|---|---|---|---|---|---|---|---|---|---|---|
| | Chin et al. (2022) [20] | Severe road-traffic trauma prediction | Taipei City Fire Department EMS and Taipei Trauma Registry, Taiwan | 2018 | 114 calls: 42 severe-trauma and 72 nonsevere cases. Repeated random subsampling validation: 104 training/10 validation cases, repeated 100 times. Cases with incomplete information were excluded; imputation: NR. Fixed class ratios were used, but no resampling was reported. | Prehospital-activated major trauma status determined by on-scene EMTs using the trauma-triage protocol | PAMT model vs six dispatchers and conventional ML models | Repeated random internal validation | Model development only | Repeated random subsampling validation, repeated 100 times; PAMT model: sensitivity 68.0%, specificity 78.0%, PPV 60.6%, NPV 85.8%, accuracy 75.0%, Youden index 0.460. AUROC and calibration: NR. | PAMT vs dispatchers: sensitivity 68.0% vs 63.1%, specificity 78.0% vs 85.0%, PPV 60.6% vs 71.7%, NPV 85.8% vs 80.3%, and accuracy 75.0% vs 76.8%. In uncertain cases, accuracy was 73.6% vs 66.3%. | The PAMT model was not superior to dispatchers overall but may assist them when they are uncertain. Larger-scale external validation is required. |

| | | | | | | | | | | | | |
|---|---|---|---|---|---|---|---|---|---|---|---|---|
| | Costa et al. (2023) [21] | Four-class emergency complaint classification | SAMU Novo Norte Emergency Regulation Center, Maringá, Paraná, Brazil | 2019 | 182,273 audio files; 10,010 manually screened and 2,326 emergency calls identified. ASR: 1,926 training, 300 evaluation, and 100 test calls. NLU training corpus: expanded to 120,000 synthetic sentences using EDA-based data augmentation; NLU test set: 587 manually classified sentences from the same SAMU dataset. Missing-data handling: NR. Class imbalance was present; no weighting | Manually produced transcripts for ASR; emergency-call categories manually assigned by trained medical students for NLU | Wav2Vec 2.0 vs HuBERT and WavLM for ASR; NLU classifier without a comparator | Internal test-set evaluation within the same study setting | Model development only | Holdout testing for ASR; independent real-world evaluation for NLU; ASR WER: Wav2Vec 2.0 42.12%, HuBERT 59.96%, WavLM 55.18%. NLU: accuracy 73.9%, precision 76.0%, recall 73.9%, F1 74.5%; category-specific AUCs 0.86–0.97. Sensitivity, specificity, and calibration: NR. | Wav2Vec 2.0 achieved the lowest WER (42.12%). The NLU model classified four emergency categories with 73.9% accuracy and 74.5% F1, with AUCs ranging from 0.86 to 0.97. | The pipeline demonstrated feasibility for Portuguese emergency-call transcription and classification, but the high transcription error and reliance on synthetic data require external real-world validation. |

| | | | | | | | | | | | | |
|---|---|---|---|---|---|---|---|---|---|---|---|---|
| | | | | | or resampling reported. | | | | | | | |
| | Ferri et al. (2021) [7] | Prediction of life-threatening level, response delay, and emergency system jurisdiction | Health Services Department medical dispatch center, Valencian Community, Spain | 2009–2012 | 1,244,624 incidents identified; 722,270 complete cases included after excluding missing labels. Design/test split: 80/20; design set further split into training/validation: 80/20. No imputation or class- | Triage labels derived from after-call data using mappings established by a 17-physician Delphi panel | DeepEMC² vs existing triage protocol and conventional ML models | Random internal holdout validation | Model development only | Random 80/20 holdout testing with an internal training/validation split; Life-threatening: recall 0.671, precision 0.742, F1 0.705, accuracy 0.771, macro F1 0.759. Response delay: macro recall 0.569, macro precision 0.589, accuracy 0.592, macro F1 0.576. Jurisdiction: | Macro F1, DeepEMC² vs protocol: 0.759 vs 0.634 (+12.5 percentage points) for life-threatening events; 0.576 vs 0.401 (+17.5 points) for response delay; 0.757 vs 0.706 (+5.1 points) for jurisdiction. Accuracy was higher by 13.2, 16.4, | In internal validation, DeepEMC² had higher performance than the existing triage protocol across all three tasks, but prospective and external validation is required. |

| | | | | | | | | | | | | |
|---|---|---|---|---|---|---|---|---|---|---|---|---|
| | | | | | resampling method reported; class imbalance was assessed using macro-averaged metrics. | | | | | recall 0.895, precision 0.827, F1 0.860, accuracy 0.801, macro F1 0.757. Specificity, AUROC, and calibration: NR. | and 4.5 percentage points, respectively. | |
| | Ferri et al. (2025) [22] | Prediction of life-threatening level, response delay, and emergency system jurisdiction | Health Services Department medical dispatch system, Valencian Community, Spain | 2009–2019, excluding 2013 | 2,054,694 incidents from 2009–2019, excluding 2013. Data were divided into seven temporal learning periods; each period used an 80/20 training/test split, with training further split 70/30 for training/validation. Missing sex was explicitly encoded; | Recorded after-call labels for life-threatening status, admissible response delay, and emergency-system jurisdiction | E2eDeepEMC$^2$ vs existing triage protocol and non-continual DeepEMC$^2$ | Temporal internal validation | Model development only | Temporal validation across seven learning periods; Average F1, current-year/next-year evaluation: life-threatening 0.675/0.660, response delay 0.583/0.569, and jurisdiction 0.804/0.793. Sensitivity, specificity, AUROC, and calibration: NR. | Compared with the existing protocol, absolute F1 gains were 18.46, 25.96, and 3.63 percentage points for life-threatening events, response delay, and jurisdiction. Gains over non-continual models were 3.04, 9.66, and 0.58 | In temporal internal validation, E2eDeepEMC$^2$ had higher F1 scores than the existing protocol and maintained similar current- and next-year performance; external validation is required. |

| | | | | | | | | | | | | |
|---|---|---|---|---|---|---|---|---|---|---|---|---|
| | | | | | other missing-data handling was NR. Class imbalance was addressed using a soft F1 loss. | | | | | | points, respectively. | |
| | Jamal Chu et al. (2021) [26] | Ambulance response time prediction for AED-delivery drone dispatch | Peel Regional Paramedic Services, Ontario, Canada | Jan 2015–Dec 2019 | 6,705 suspected OHCAs identified; 3,573 included after excluding incomplete or erroneous location and response-time data. Five-fold cross-validation (80/20) was used. Imputation: NR. | Recorded ambulance response time; calculated drone response time treated as the reference for simulated dispatch decisions | ML-based selective drone dispatch vs always-dispatch and never-dispatch policies | Cross-validation with simulation-based evaluation | Simulation only | Five-fold cross-validation; Representative linear-regression rule (δ=0): sensitivity 93.8%, specificity 76.0%, accuracy 87.8%. Neural-network rule (δ=0): sensitivity 93.5%, specificity 77.1%, accuracy 87.9%. Response-time prediction errors and calibration: NR. | ML-based selective dispatch was estimated to reduce median response time from 5.8 to 3.9 min and mean response time from 6.2 to 4.1–4.2 min (all $p<0.001$). It was noninferior to always dispatching ($p<0.001$) while reducing | Simulation-based estimates indicated that selective ML-guided drone dispatch could achieve response-time reductions comparable to universal drone dispatch while requiring |

| | | | | | | | | | | | | |
|---|---|---|---|---|---|---|---|---|---|---|---|---|
| | | | | | | | | | | | drone flights by up to 30%. | fewer flights; real-world validation is required. |
| | Salah Taamneh et al. (2021) [23] | Road-traffic injury severity prediction | Abu Dhabi Emirate road-traffic accident system, United Arab Emirates | 2008–2013 | 5,973 accidents; 5,520 evaluated using 10-fold cross-validation. Imbalance was addressed using a cost matrix. Missing-data handling: NR. | Injury-severity category recorded in the accident dataset and validated using information from dispatched medical units | Cost-sensitive ANN; no comparator reported | Cross-validation | Model development only | Ten-fold cross-validation; Accuracy: 65.1%. Sensitivity/specificity: death 69.2%/97.6%, severe 62.2%/96.5%, moderate 62.6%/74.1%, minor 66.4%/71.7%. Numerical AUC and calibration: NR. | Correctly classified 3,594/5,520 cases (65.1%), with the highest specificity for death (97.6%) and severe injury (96.5%). | The model showed moderate accuracy and high specificity for serious injuries, but lacked a comparator and external validation. |
| | Selvan et al. (2025) [27] | Real-time ambulance route optimization | Specific EMS setting not reported | NR | Kaggle dataset comprising 605 files; training/test split: 80/20. Missing-data handling: NR. | NR; the derivation of the optimal-route labels was unclear | Proposed CRNN route model vs DRL, PPO, ADL, and LLM models | Random internal holdout validation | Model development only | Random 80/20 holdout; reported accuracy 92.3%, precision 91.0%, recall 90.8%, F1 90.8%, and computation time 720 ms. AUROC and calibration: NR. | The proposed model had higher reported metrics and shorter computation time than the comparator models. | The authors concluded that the model could support ambulance route optimization; however, |

| | | | | | | | | | | | | |
|---|---|---|---|---|---|---|---|---|---|---|---|---|
| | | | | | | | | | | | | unclear prediction targets and reference-route labels limit interpretation of the reported metrics. |
| | Wang et al. (2023) [24] | Three-level acuity classification | Singapore Civil Defence Force national emergency call center, Singapore | Jan 2018–Aug 2020 | 361,506 cases; 10 random training/test splits of 80/20. Class distribution: 54,134 critical, 233,886 emergency, and 73,486 nonemergency cases. Missing age was median-imputed; unanswered categorical variables were coded as “not asked.” Class weights were optimized to control under-triage. | Patient Acuity Category Scale recorded by paramedics at the scene | Random forest vs call-center specialists and conventional ML models | Repeated random internal holdout validation | Model development only | Ten repeated random 80/20 holdout splits; Random forest: accuracy 60.8% (95% CI 60.7–60.9), over-triage 31.1% (31.0–31.2), under-triage 8.1% (8.0–8.1), critical likelihood ratio 3.2, and nonemergency likelihood ratio 5.3. Sensitivity, specificity, AUROC, and calibration: NR. | Random forest vs specialists: accuracy 60.8% vs 47.6%, over-triage 31.1% vs 45.7%, under-triage 8.1% vs 6.7%, critical likelihood ratio 3.2 vs 1.8, and nonemergency likelihood ratio 5.3 vs 2.7. | In repeated internal validation, the model had lower over-triage and higher accuracy, but higher under-triage, than call-center specialists. |

| | | | | | | | | | | | | |
|---|---|---|---|---|---|---|---|---|---|---|---|---|
| | Wibring et al. (2022) [8] | High- and low-risk prediction in patients with chest pain | SOS Alarm emergency medical dispatch center and regional EMS system, Sweden | 2018 | 2,917 EMS missions; training/validation split: 2,049/868 (70/30). Missing data were imputed using MissForest before data splitting. Validation set: high-risk 17%, intermediate-risk 15%, low-risk 68%. | Final hospital-discharge diagnosis assigned by the responsible physician | Logistic regression models vs the existing SIMPD triage protocol | Random internal holdout validation | Model development only | Random 70/30 holdout validation; High-risk model: AUROC 0.789, sensitivity 89%, specificity 51%, PPV 27%, NPV 96%. Low-risk model: AUROC 0.737, sensitivity 10%, specificity 97%, PPV 89%, NPV 33%. Calibration: NR. | High-risk model vs SIMPD: sensitivity 89% vs 77%, specificity 51% vs 38%, PPV 27% vs 20%, NPV 96% vs 89%; highest-priority dispatch 56% vs 65%. Low-risk model vs SIMPD: sensitivity 10% vs 1%, specificity 97% vs 99%, PPV 89% vs 64%, NPV 33% vs 32%; lowest-priority assignment 7% vs 1%. Both comparisons: $p < 0.001$. | In internal validation, the models had higher selected performance measures than the SIMPD protocol and assigned fewer cases to the highest dispatch priority. |

| | | | | | | | | | | | | |
|---|---|---|---|---|---|---|---|---|---|---|---|---|
| Scenario- or simulation-based evaluation | Li et al. (2026) [30] | Simulation of emergency medical dispatcher–caller interactions | No operational EMS setting | NA | 100 simulated dispatch cases covering 32 chief complaints and six caller profiles; evaluated by four physicians. Twenty cases were rated by all four physicians. | No independent reference standard; simulated outputs were evaluated by four physicians using a taxonomy-based assessment framework | Taxonomy-grounded LLM multi-agent system (DispatchMAS); no direct baseline comparator | Simulation-based evaluation | Simulation only | Inter-rater agreement: Gwet's AC1 >0.70. Correct external-agent contact: 94%; callback instructions: 97%; advice provided when needed: 91%. Dispatcher language: 73.7% neutral sentiment, 90.4% neutral emotion, Flesch readability 80.9, and 60% polite/0% impolite. Calibration: NA. | High ratings were reported for caller-role alignment (96.3%), resistance management (96.3%), shared problem framing (87.5%), explanation of questioning (87.5%), and establishing the caller's knowledge basis (90.0%). Mean simulated response time was 1.8 s for life-critical events versus 2.1–2.4 s for other cases. | DispatchMAS generated clinically plausible and protocol-aligned simulated calls, but validation using real emergency calls and comparison with existing dispatch systems are required. |

| | | | | | | | | | | | | |
|---|---|---|---|---|---|---|---|---|---|---|---|---|
| | Nun et al. (2025) [31] | Emergency medical dispatch dialogue dataset construction | CHU Avicenne and CHU La Réunion, France | 2022–2023 | 61 simulated dispatch calls from 17 junior doctors; 3 h 14 min; 2,997 utterances. Leave-one-speaker-out cross-validation for in-domain fine-tuning. | Manually prepared transcripts and speaker annotations | SIMSAMU-fine-tuned CTC-wav2vec vs. ESLO2-fine-tuned CTC-wav2vec and Whisper; Pyannote diarization evaluated separately | Leave-one-speaker-out cross-validation | Simulation/dataset evaluation only | WER: 16.1% (SIMSAMU-fine-tuned CTC), 24.8% (ESLO2-fine-tuned CTC), and 35.8% (Whisper). CER: 10.7%, 16.6%, and 27.8%, respectively. DER: 10.4%. Calibration: NA. | In-domain fine-tuning was associated with a lower WER (16.1% vs 24.8%). Four caller and three dispatcher utterance clusters were identified, with two principal dialogue phases: interrogation and contractualization. | SIMSAMU provides an open benchmark for French medical-dispatch dialogue processing, and domain-specific fine-tuning substantially improved speech recognition. |
| | Shekhar et al. (2025) [28] | Selection of the highest-priority request type among four options | Major metropolitan EMS system in the United States; city not reported | NR | 392 ambulance requests arranged into 98 groups of four | Consensus or majority decision of three experienced critical-care paramedics | Zero-shot GPT-4o Mini prioritization vs. decisions by three experienced critical-care paramedics | Scenario-based evaluation | Simulation only | Overall agreement: 76.5% (75/98); agreement for unanimous panel decisions: 93.8% (45/48); agreement for majority decisions: 68.2% (30/44). Calibration: NA. | Panel decisions: unanimous, 48/98; majority, 44/98; split, 6/98. In 9/14 disagreements with a panel majority, the LLM selected the minority | GPT-4o Mini showed promising prioritization ability, particularly when experts agreed, but the findings remain preliminary. |

| | | | | | | | | | | | | |
|---|---|---|---|---|---|---|---|---|---|---|---|---|
| | | | | | | | | | | | paramedic decision. | |

ADL, adaptive deep learning; AED, automated external defibrillator; AI, artificial intelligence; ANN, artificial neural network; ASR, automatic speech recognition; AUC, area under the curve; AUROC, area under the receiver operating characteristic curve; CER, character error rate; CI, confidence interval; CPR, cardiopulmonary resuscitation; CRNN, convolutional recurrent neural network; CTC, connectionist temporal classification; DER, diarization error rate; DRL, deep reinforcement learning; EDA, easy data augmentation; EMD, emergency medical dispatch; EMS, emergency medical services; FN, false negative; FP, false positive; FPR, false-positive rate; HuBERT, Hidden-Unit BERT; k-NN, k-nearest neighbors; LLM, large language model; ML, machine learning; MPDS, Medical Priority Dispatch System; NA, not applicable; NLU, natural language understanding; NPV, negative predictive value; NR, not reported; OHCA, out-of-hospital cardiac arrest; OR, odds ratio; PAMT, prehospital-activated major trauma; PPO, proximal policy optimization; PPV, positive predictive value; RCS, resource-constrained situation; SIMPD, Swedish Index for Medical Priority Dispatch; SIMSAMU, a French medical dispatch dialog open dataset; SVM, support vector machine; TF-IDF, term frequency–inverse document frequency; TN, true negative; TP, true positive; WER, word error rate.

**Table S3. Quality assessment of the included studies**

**Table S3a. Risk-of-Bias Assessment of Randomized Controlled Trials Using Cochrane RoB 2**

| Author (Year) | Assessed outcome | D1: Randomization process | D2: Deviations from intended interventions | D3: Missing outcome data | D4: Measurement of the outcome | D5: Selection of the reported result | Overall risk of bias |
|---|---|---|---|---|---|---|---|
| Blomberg et al.(2021) [6] | Dispatcher recognition rate of subsequently confirmed OHCA | Low | Some concerns | Low | Low | Some concerns | Some concerns |
| Spangler et al. (2026) [29] | Proportion of resource-constrained situations in which the first available ambulance was dispatched to the patient with the highest NEWS | Some concerns | High | High | Low | Some concerns | High |

D, domain; NEWS, National Early Warning Score; OHCA, out-of-hospital cardiac arrest; RoB 2, revised Cochrane risk-of-bias tool for randomized trials.
Low, Some concerns, and High indicate low risk of bias, some concerns regarding risk of bias, and high risk of bias, respectively.

**Table S3b. Risk-of-Bias and Applicability Assessment of AI Diagnostic Accuracy Studies Using QUADAS-3**

| Author (Year) | Accuracy Estimate Assessed | D1: Participants | D2: Index Test | D3: Target Condition | D4: Analysis | Overall Risk of Bias | Applicability: Participants | Applicability: Index Test | Applicability: Target Condition | Overall Concern Regarding Applicability |
|---|---|---|---|---|---|---|---|---|---|---|
| Blomberg et al. (2023) [18] | ML-based OHCA detection in routine clinical deployment | Low | Low | Low | High | High | Low | Low | Low | Low |
| Byrsell et al. (2021) [19] | ML-based OHCA detection at the prespecified threshold | High | Low | High | High | High | High | Low | Low | High |

AI, artificial intelligence; D, domain; ML, machine learning; OHCA, out-of-hospital cardiac arrest; QUADAS-3, Quality Assessment of Diagnostic Accuracy Studies, version 3
For risk-of-bias domains, Low, High, and Insufficient information indicate low risk, high risk, and insufficient information to judge risk of bias, respectively. For applicability domains, Low, High, and Insufficient information indicate low concern, high concern, and insufficient information to judge applicability, respectively.

**Table S3c. Assessment of AI-Based Prediction Model Development and Evaluation Studies Using PROBAST+AI**

| Author (Year) | Prediction model/outcome assessed | D1: Participants and Data Sources | D2: Predictors | D3: Outcome | D4: Analysis | Overall Risk of Bias | Applicability: Participants and Data Sources | Applicability: Predictors | Applicability: Outcome | Overall Concern Regarding Applicability |
|---|---|---|---|---|---|---|---|---|---|---|
| Anthony et al. (2021) [17] | Classification of sepsis, myocardial infarction, and cardiac arrest from caller descriptions | High | Low | Unclear | High | High | Low | Low | Low | Low |
| Ceklic et al. (2022) [25] | Prediction of the need for a lights-and-sirens response to traffic crashes | Low | Low | Unclear | High | High | Low | Low | Low | Low |
| Chin et al. (2022) [20] | Prediction of severely injured patients using emergency-dispatch text | Low | Low | Low | High | High | Low | Low | Low | Low |
| Costa et al. (2023) [21] | Four-class emergency-call classification using speech models | High | Low | High | High | High | Unclear | Low | High | High |
| Ferri et al. (2021) [7] | DeepEMC²: life-threatening level, response delay, and EMS jurisdiction | High | Low | Unclear | High | High | Low | Low | Low | Low |
| Ferri et al. (2025) [22] | Multimodal continual-learning prediction of life-threatening level, response delay, and EMS jurisdiction | Low | Low | Unclear | High | High | Low | Low | Low | Low |
| Jamal Chu et al. (2021) [26] | Prediction of ambulance response time for AED-drone dispatch | Low | Low | Low | High | High | Low | Low | Low | Low |
| Salah Taamneh et al. (2021) [23] | ANN prediction of road-traffic injury severity | High | Unclear | Unclear | High | High | Unclear | Unclear | Low | Unclear |
| Selvan et al. (2025) [27] | Real-time ambulance-route optimization using DNN/CRNN | Unclear | Low | Unclear | High | High | High | Low | High | High |
| Wang et al. (2023) [24] | Three-level ambulance-dispatch acuity classification | Low | Low | Low | High | High | Low | Low | Low | Low |
| Wibring et al. (2022) [8] | High- and low-risk prediction among patients with chest pain | Low | High | Low | High | High | Low | High | Low | High |

AED, automated external defibrillator; AI, artificial intelligence; ANN, artificial neural network; CRNN, convolutional recurrent neural network; D, domain; DeepEMC², deep ensemble multitask classifier for emergency medical calls; DNN, deep neural network; EMS, emergency medical services; PROBAST+AI,

Prediction Model Risk of Bias Assessment Tool for Artificial Intelligence

**Table S3d. Methodological Assessment of the Agreement Study Using an Adapted QAREL Framework**

| Author (Year) | Agreement Construct Assessed | QAREL Item-Level Findings | Key Methodological Limitations | Applicability Limitations |
|---|---|---|---|---|
| Shekhar et al. (2025) [28] | LLM–paramedic agreement on ambulance dispatch prioritization | 3 Yes, 3 No, 2 Unclear, and 3 N/A | Constructed scenarios, small expert panel, no independent reference standard, incomplete LLM settings, and limited agreement statistics | No evaluation using actual EMS calls or routine clinical and operational outcomes |

EMS, emergency medical services; LLM, large language model; N/A, not applicable; QAREL, Quality Appraisal of Reliability Studies

QAREL item-level findings were categorized as Yes, No, Unclear, or N/A. Methodological and applicability limitations were summarized descriptively; no numerical score or overall QAREL rating was assigned.
